# Potential repurposing of DPP4 inhibitors for target therapy resistance in renal cell carcinoma

**DOI:** 10.18632/oncotarget.28463

**Published:** 2023-09-15

**Authors:** Kuniko Horie, Satoshi Inoue

**Keywords:** renal cell carcinoma (RCC), tyrosine kinase inhibitor (TKI), Dipeptidyl peptidase IV (DPP4), drug resistance, drug repurposing

Renal cell carcinoma (RCC) is a major adult kidney cancer, which is often incidentally discovered as an asymptomatic disease on imaging in the developed countries [1]. RCC has the most fatal disease among urological cancers, as a recent 5-year relative survival rate in US (2009–2015) is less than 80% [1]. While RCC is known as a cancer resistant to chemo- and radio-therapies, the prognosis of RCC has been remarkably improved after the clinical application of tyrosine kinase inhibitors (TKIs) and immunotherapy [2]. The rationale for the efficacy of TKIs in RCC is mainly based on the angiogenetic status, particularly in clear cell RCC (ccRCC) that is the most common type of RCC (70–75% of RCC) [3], in which the loss of function mutation of Von Hippel-Lindau (*VHL*) tumor suppressor gene activates hypoxia inducible factor (HIF) and vascular endothelial growth factor (VEGF) pathways. The first-line TKIs that predominantly target VEGF receptor (VEGFR) and platelet-derived growth factor receptor (PDGFR) (e.g., sunitinib and sorafenib) have been clinically used since late 2000s, and the second-line TKIs such as cabozantinib, which targets more receptor tyrosine kinases including MET and TAM kinases as well as VEGFR, have been further applied to the treatment of advanced RCC since early 2010s in which the first-line TKIs are ineffective. The mTOR (mechanistic target of rapamycin) protein kinase pathway is also a crucial axis involved in the pathogenesis of RCC including both ccRCC and non-ccRCC, the latter mainly consists of papillary and chromophobe types of RCC, and mTOR inhibitors everolimus and temsirolimus are applied to the treatment of non-ccRCC [4]. While the recent application of immune checkpoint blockage (ICB), targeting programmed cell death 1 PD-1 or its ligand PD-L1 and cytotoxic T lymphocyte antigen 4 (CTLA-4), has changed the strategies against advanced RCC, TKIs or mTOR inhibitors are used in combined treatment with ICB or in monotherapies, especially for patients who are intolerable to receive immunotherapy [2]. Therefore, the overcome of target therapy resistance against protein kinase inhibitors remains as a critical issue for patients with advanced RCC.

While several molecular mechanisms underlying TKI resistance have been characterized, the oncogenic roles of proinflammatory cytokines interleukin-6 (IL6) and IL8 are of particular interest as prognostic factors, particularly in ccRCC [5]. The secretion of IL6 has been shown to further activate mTOR and STAT3 signaling, as well as VEGF pathway, and the inhibition of IL6 and IL8 recovers the TKI sensitivity in TKI-resistant RCC cells [5]. The activation of these cytokines is also associated with stem-like phenotypes of RCC cells, with the elevated expression of stem cell markers such as CD44, CD133, CXC-chemokine receptor 4 (CXCR4), and aldehyde dehydrogenase 1 (ALDH1) [6], because IL6 induces cancer stemness and epithelial-to-mesenchymal transition (EMT) by upregulating STAT3 and ZEB2 and subsequently activating stem cell markers [7]. Dipeptidyl peptidase IV (DPPIV), an intrinsic type II transmembrane glycoprotein and a serine exopeptidase, has been defined as another stem cell marker in cancers with tumor-initiating phenotypes and metastasis [8]. In our recent study, we established a panel of patient-derived ccRCC spheroid cultures with the enhancement of cancer stemness gene signature including DPP4 [9]. Focusing on TKI sunitinib sensitivity, we demonstrated that DPP4 inhibition increased sunitinib efficacy in DPP4-high RCC spheroids and DPP4 was upregulated in sunitinib-resistant RCC cells. DPP4 inhibitors (DPP4is) such as sitagliptin are clinically used in the treatment of type 2 diabetes millitus (T2DM) and DPP4i administration for RCC patients with T2DM potentially improves tumor-suppressive responses of TKI and prognoses of patients with TKI therapy. The prognostic analysis of clinical RCC cases is based on a limited number of patients, thus, a clinical study with a larger number of RCC patients will further demonstrate the TKI-facilitating efficacy of DPP4i. Nevertheless, DPP4i sitagliptin substantially reduced the *in vivo* tumor formation of sunitinib-resistant RCC cells in mice, suggesting the TKI-facilitating effects of DPP4i in RCC pathophysiology.
